# Survival time of *Leptospira kirschneri* serovar Grippotyphosa under different environmental conditions

**DOI:** 10.1371/journal.pone.0236007

**Published:** 2020-07-15

**Authors:** L. H. Nau, A. Obiegala, N. Król, A. Mayer-Scholl, M. Pfeffer

**Affiliations:** 1 Institute of Animal Hygiene and Veterinary Public Health, University of Leipzig, Leipzig, Germany; 2 Department Biological Safety, German Federal Institute for Risk Assessment, Berlin, Germany; UFPL, BRAZIL

## Abstract

Leptospirosis is a re-emerging zoonotic disease of high medical importance that affects humans worldwide. Humans or animals acquire an infection with pathogenic leptospires either by direct contact with infected animals or by indirect contact to contaminated environment. Survival of *Leptospira* spp. in the environment after having been shed via animal urine is thus a key factor to estimate the risk of infection, but not much is known about the tenacity of pathogenic leptospires. Here, the survival time of both a laboratory strain and a field strain of *L*. *kirschneri* serovar Grippotyphosa in animal urine and their tenacity while drying was investigated and compared at different temperatures (15°C-37°C). *Leptospira* spp. are also often found in rivers and ponds. As the infection risk for humans and animals also depends on the spreading and survival of *Leptospira* spp. in these environments, the survival of *L*. *kirschneri* serovar Grippotyphosa was investigated using a 50-meter-long hose system simulating a water stream. Both strains did not survive in undiluted cattle or dog urine. Comparing different temperatures and dilution media, the laboratory strain survived the longest in diluted cattle urine with a slightly alkaline pH value (3 days), whilst the field strain survived in diluted dog urine with a slightly acid pH value up to a maximum of 24 h. Both strains did not survive drying on a solid surface. In a water stream, leptospires were able to move faster or slower than the average velocity of the water due to their intrinsic mobility but were not able to survive the mechanical damage caused by running water in the hose system. From our results we conclude, that once excreted via animal urine, the leptospires immediately need moisture or a water body to survive and stay infectious.

## Introduction

Pathogenic spirochetes of the genus *Leptospira* are the cause of a febrile zoonosis called leptospirosis which affects approximately more than 1 million people annually [1]. Although the risk of becoming infected is highest in tropical and subtropical regions, there are also around 24,000 human cases per year in Europe, with 5% having a fatal outcome [1]. The infection may manifest in humans with a high variability of symptoms, ranging from subclinical infections over mild often flu-like symptoms to severe illnesses with signs of multi-organ dysfunction. Due to unspecific clinical signs, a very large number of undetected leptospiral infections is assumed [2]. Symptoms of animals with leptospirosis are also very variable and depend not only on the species but also on the constitution, age and leptospiral serovar. Infections of adult livestock like cattle or swine with leptospirosis have a major economic significance by causing various forms of reproductive failure [3].

*Leptospira* spp. are small (6–20 μm long with an average diameter of 0.1 μm), highly motile, spiral-shaped bacteria with hooked ends [4, 5]. There are two taxonomic classification systems of *Leptospira* spp., one based on serological features dividing the pathogenic *Leptospira* spp. into 24 serogroups with at least 300 serovars and the other based on differences in the genome distinguishing currently 65 *Leptospira* species. [6–8]. Although the two classification systems are not congruent, for example, serovars of the same serogroup can be assigned to different genome species, both are used today [8–10]. *Leptospira kirschneri* serovar Grippotyphosa is one of the most important infectious agents associated with human leptospirosis outbreaks in Germany [11, 12]. Additionally, *Leptospira* spp. of the serogroup Grippotyphosa are one of the main causes of canine leptospirosis in Europe and can also be found in many other mammals like cattle, swine, sheep, horses and rodents [2, 13–15]. As these animals are often living in close proximity to humans their infections do not only play an economic role but are also important for the assessment of the human infection risk. Rodents and other small mammals are well known reservoir hosts for *Leptospira* and play a major role in the dissemination of many leptospiral species [16, 17].

Humans usually get infected with *Leptospira* spp. through direct contact with an infected animal or through indirect contact with the urine of these animals via contaminated soil or water. Mucous membranes, abrasions or cuts in the skin are usual portals of entry [2].

Thus, the survival of *Leptospira* spp. in the environment is a crucial factor influencing the risk of infection for humans and animals. The survival time of *Leptospira* spp. in the environment depends on various factors, for example, the pH value of the urine, the temperature of the environment, UV radiation, the leptospiral strain, the type of surface the leptospires are excreted on (water, soil, etc.), and its chemical and bacterial composition [18–23].

There are many reports of *Leptospira* spp. found in rivers or creeks and outbreaks of human leptospirosis acquired by contact to water sources [24–26]. Therefore, also the distribution and the survival of the pathogen in water streams are of importance to estimate the risk of human infections.

Thus far, examinations of the survival time of *Leptospira* spp. in the environment have been scarce despite its importance to human leptospiral infections. Existing studies mostly consider the survival time of *Leptospira* spp. in different types of soil or water (under various conditions) [18, 19, 22, 24]. There are only very few studies examining the survival time of *Leptospira* spp. in animal urine ex vivo or its distribution in the environment [18, 27]. To our knowledge, no research so far has compared the survival of these bacteria in different kinds of animal urine and the possible differences in survival time between a laboratory and a field strain.

The achieved aims of the study were to examine the survival time of *Leptospira kirschneri* (a laboratory and a field strain) outside the host under different conditions imitating possible environmental scenarios: (1) its survival in dog and cattle urine, (2) the effect of dilution after the excretion, (3) its distribution and survival in a water stream, and (4) its tenacity while drying.

## Material and methods

### Leptospiral isolates

The leptospiral isolates used in this study were obtained from the German national consultant laboratory for leptospirosis at the Federal Institute for Risk Assessment in Berlin. The choice of *L*. *kirschneri* serovar Grippotyphosa was based on its frequent appearance and its detection in two recent (2007 and 2014) human outbreaks of leptospirosis in Germany [11, 12]. Leptospiral strains were maintained at room temperature in liquid Ellinghausen and McCullough medium (EMJH) as modified by Johnson and Harris (BD-Difco EMJH Medium Base and Enrichment, Thermo Fisher Scientific, Schwerte, Germany) [28]. Subcultures were incubated in liquid EMJH medium at 29°C for 3–4 days and then stored at 23°C in the dark until use. The number of viable organisms per ml of culture was analyzed using a Thoma cell counting chamber (0.1 mm depth). All experiments were conducted with a one-week-old laboratory strain of *L*. *kirschneri* serovar Grippotyphosa (Strain Moska, passages 93–97) and a field strain of *L*. *kirschneri* serovar Grippotyphosa (LA1-RoBo-Pub, passages 5–8) isolated in 2017. Mechanical damage and UV-light exposure of the leptospires during the handling was avoided as much as possible.

### Collection and processing of urine samples

Because both cattle and dogs can be infected with *L*. *kirschneri* serovar Grippotyphosa, their urine was selected for the experiments. Furthermore, these animals were chosen as there are interesting differences between their urine, e.g. in pH values and biochemical composition, exemplifying the urine of herbivore and carnivore species. Urine samples were collected as middle stream samples from healthy untreated animals, which showed no signs of leptospirosis. The cattle urine was obtained from cows of the Clinic for Ruminants and Swine of the University of Leipzig. The dog urine was obtained from one privately kept pet dog (9 years old, male Magyar Vizsla). After collection, the urine was sterile filtrated and kept frozen in aliquots at -20°C until usage. The animals urinated spontaneously and the urine was only used as matrix and not investigated further. Therefore, no ethical approval was necessary. Neither cattle nor the dog were manipulated in any way during urine collection. As the dog’s owner is part of the Institute of Animal Hygiene and Veterinary Public Health in Leipzig and volunteered to provide the dog’s urine for the experiments no further consent was necessary.

### DNA-extraction and real-time PCR

DNA was extracted from samples using the commercial QIAamp DNA Mini Kit (Qiagen, Hilden, Germany). A real-time PCR targeting the *Lipl32* gene was conducted according to Stoddard et al. [29] with slight modifications (as described by Woll et al. [30] without the use of the internal control).

### Survival of *L*. *kirschneri* serovar Grippotyphosa in animal urine

The experiments were conducted with undiluted and diluted urine. Dilution (1:10) was done either in phosphate-buffered saline (PBS) or in purified water. Then, 225 μl of urine (diluted or undiluted) was placed in 96-well-plates and 25 μl of a leptospiral culture was added (field strain with a concentration of 4.7–5.5 x 10^7^ bacteria/ml; laboratory strain with a concentration of 3.1–3.53 x 10^8^ bacteria/ml). After sample incubation at 15°C, 23°C, 29°C, and 37°C for different periods (1 min, 5 min, 10 min, 30 min, 1 h, 2 h, 4 h, 24 h, and then daily until day 7), a 200 μl aliquot was taken from each well and added to 4 ml of fresh EMJH medium. The cultures were then incubated at 29°C for at least 28 days and checked weekly for motile *Leptospira* under the dark field microscope. All experiments were conducted in triplicate.

As a positive growth control during each experiment, the same number of leptospires was put into 225 μl PBS and incubated for 1 min and 24 h at four different temperatures (15°C, 23°C, 29°C, and 37°C). These controls were handled and tested for leptospiral growth exactly as described for the urine samples. Controls were done for both strains at all tested temperatures.

Furthermore, to prove that failed cultivation attempts did not arise from negative influences of PBS or handling, cultures of *L*. *kirschneri* serovar Grippotyphosa (10^8^ leptospires/ml) in EMJH medium were stored at 15°C, 23°C, 29°C, and 37°C for one week. After 7 days, 200 μl of culture was taken from each tube and put into 4 ml EMJH medium. The tubes were incubated at 29°C and checked for leptospiral growth.

### Distribution and survival of *L*. *kirschneri* serovar Grippotyphosa in a water stream

**Fig 1:**
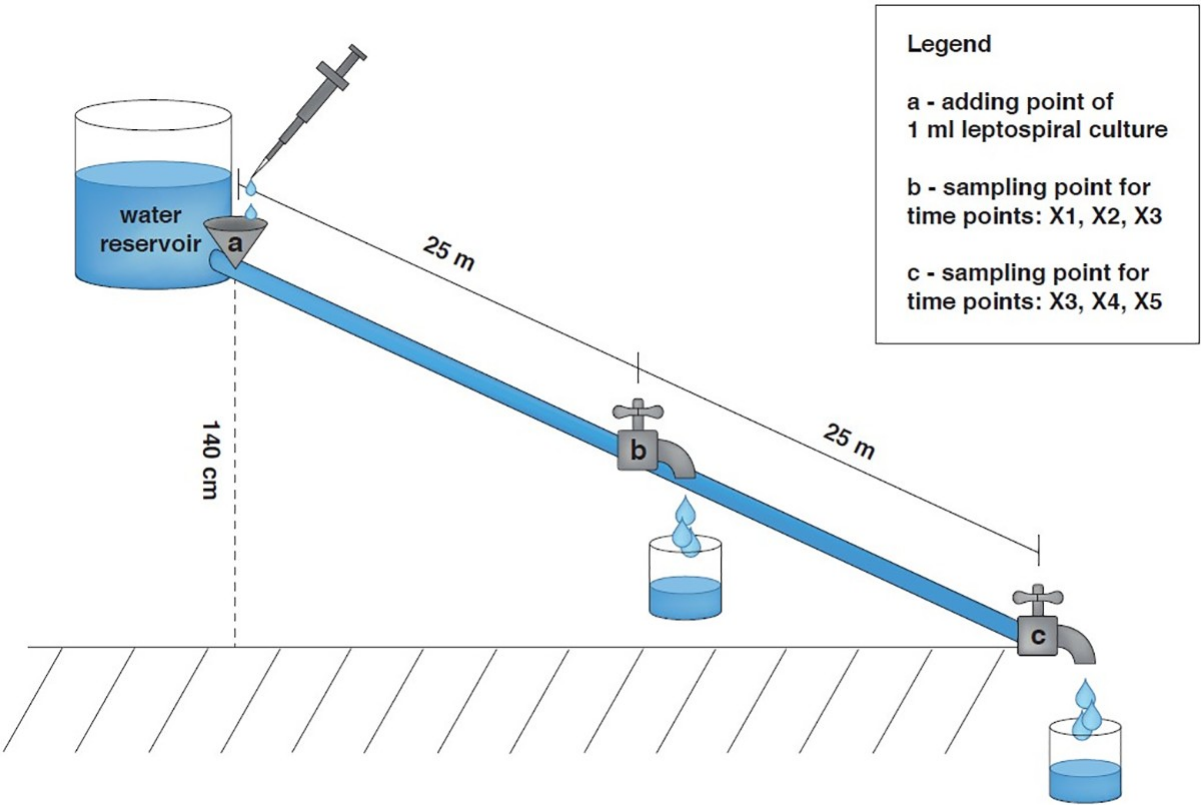
Schematic overview of the 50-meter-long hose system. Between point a and c there is an altitude difference of 140 cm. Time points for taking water samples: X1: 37 min 30 sec; X2: 41 min 36 sec; X3: 45 min 48 sec; X4: 79 min 12 sec; X5: 83 min 18 sec; X6: 87 min 30 sec.

In order to investigate the distribution and the survival of *L*. *kirschneri* serovar Grippotyphosa in rivers or creeks, a water stream system containing a water reservoir filled with tap water and a 50-meter-long hose with a point for adding leptospiral cultures was built (Fig 1). The hose system had two outlet valves: one after 25 m (Fig 1, point b) and the second after 50 m (Fig 1, point c) for the withdrawal of samples. A constant flow velocity of 0.01 m/sec on average was generated by regulating the water efflux at the endpoint of the system and an elevation difference of 140 cm between the beginning and the end of the tube.

As the speed of leptospires due to their intrinsic mobility in the water stream was not predictable, samples were taken after different time intervals at the two mentioned outlet valves (Fig 1: time points: X1, X2, X3, and respectively X4, X5, X6). The time points X1 (37 min 30 sec) and X4 (79 min 12 sec) were chosen due to the assumption that leptospires moved faster than the regular water stream, X2 (41 min 36 sec) and X5 (83 min 18 sec) that they were as fast as the water stream, and points X3 (45 min 48 sec) and X6 (87 min 30 sec) presuming leptospires moved slower than the regular water stream because of their intrinsic mobility in the opposite direction. For each partial experiment, 1 ml leptospiral culture in EMJH medium (containing 2.1 x 10^8^–3.6 x 10^8^ leptospires) was added into the hose system (Fig 1, point a) filled with tap water. At all time points (X1 –X6), 2 ml of water was carefully withdrawn at the sampling point b or c. After filtration through filters (pore size 0.22 μm, Sarstedt, Nümbrecht, Deutschland), to eliminate contamination, 200 μl of sample was taken for an examination by real-time PCR and 400 μl of each sample was added to 4 ml of modified EMJH medium. The tubes were incubated at 29°C for at least 28 days and checked weekly for motile leptospires under the dark field microscope. Each experiment was conducted for the field and laboratory strain.

As a control, 500 μl of the same leptospiral cultures used for the water stream experiment was added into 20 ml stagnant tap water and left at room temperature for at least two hours. Afterwards, samples were taken, filtrated and handled identically as the samples taken from the hose system and cultivation attempts were done in triplicate. This step was conducted to prove that any failed attempts of cultivation did not arise from the damage of the leptospires due to the contents of the tap water or during filtration but only due to mechanical damage during passage in the hose system.

### Stability of *L*. *kirschneri* serovar Grippotyphosa while drying

To determine the stability of the different strains of *L*. *kirschneri* serovar Grippotyphosa against drying, 50 μl of leptospiral culture (containing 7.2 x 10^7^–7.9 x 10^7^ bacteria/ml) was placed on sterilized steel discs (20 mm in diameter) and left to dry up at 15°C, 23°C, 29°C, and 37°C. Every 30 min the discs were checked for their drying condition. The time point “completely dry” was defined as the time when there was no liquid visible on the steel discs and the time point “almost dry” was defined as the last time point tested before complete drying, i.e. 30 min less. The discs were rinsed off with 1 ml of modified EMJH medium and added into tubes with 3 ml of EMJH medium and incubated at 29°C. For at least 28 days the tubes were checked weekly under a dark field microscope for the appearance of motile *Leptospira*. All experiments were conducted in triplicate.

**Fig 2:**
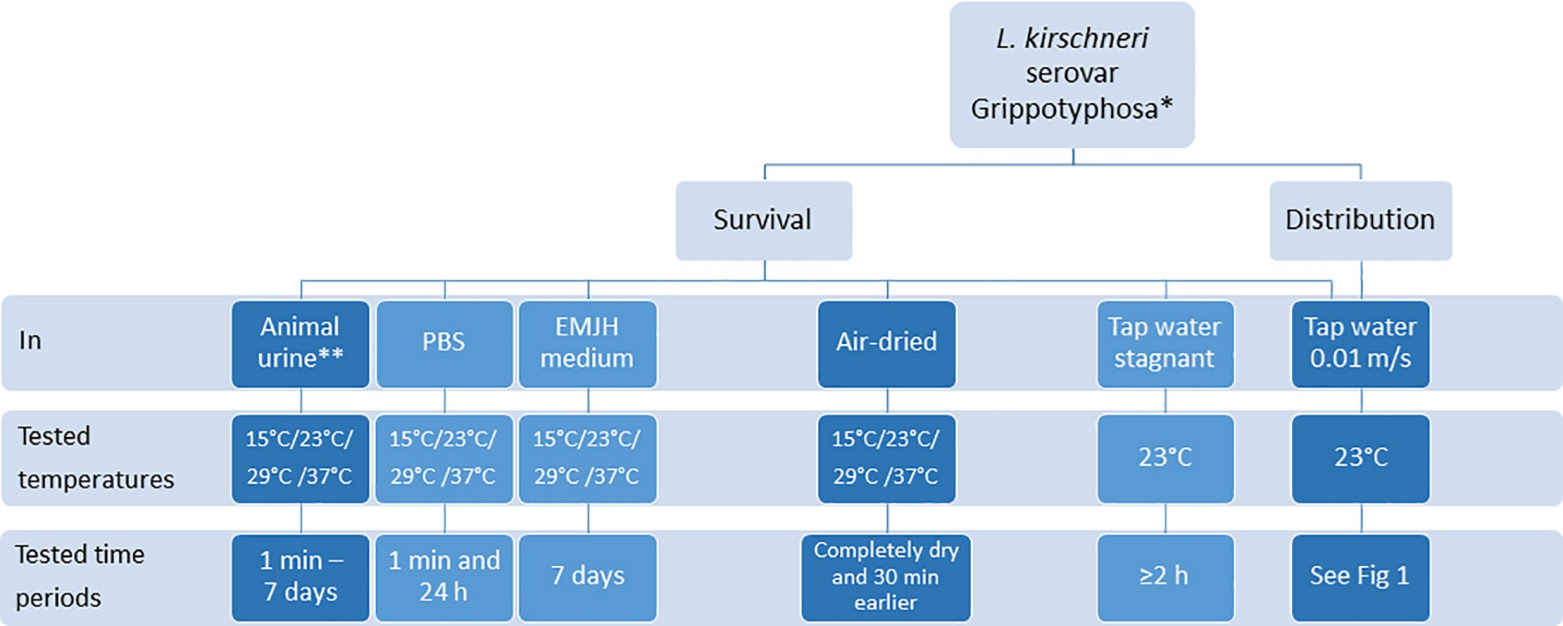
Schematic flowchart of the study design. * laboratory strain or field strain. ** cattle or dog urine, undiluted or diluted 1:10 in either PBS or purified water. Experiments shown in dark blue; controls shown in light blue.

Fig 2 shows a flowchart of the study design for all experiments conducted with *L*. *kirschneri* serovar Grippotyphosa.

### Statistical analysis

The two-tailed Mann-Whitney-U test was used to determine differences in the survival time comparing incubation temperatures, media in which *L*. *kirschneri* serovar Grippotyphosa was diluted in, and different leptospiral strains with a standard error (α = 0.05) (IBM SPSS Statistics 25, Armonk, New York, United States).

## Results

### Survival of *L*. *kirschneri* serovar Grippotyphosa in animal urine

In diluted urine, both strains of *L*. *kirschneri* serovar Grippotyphosa were able to survive for a period between 1 hour and three days (see Table 1 for all survival times). The laboratory strain survived significantly longer in diluted cattle urine compared to the field strain (p = 0.027), while the field strain survived significantly longer than the laboratory strain in diluted dog urine (p = 0.028). Both strains had the shortest survival time in cattle urine diluted in PBS at 37°C. The survival time of the laboratory strain in cattle urine diluted with PBS was significantly longer than in urine diluted with distilled water (p = 0.039). In contrast, the field strain survived significantly longer in cattle or dog urine which was diluted in distilled water (p = 0.013, p = 0.013, respectively). The temperature was a crucial influencing factor as the field, as well as the laboratory strain, survived significantly longer at 15°C temperature when compared to 37°C (p <0.001, p = 0.041, respectively). Neither the laboratory nor the field strain of *L*. *kirschneri* serovar Grippotyphosa survived in undiluted dog or cattle urine at any examined temperature.

**Table 1:** Survival time of *Leptospira kirschneri* serovar Grippotyphosa (field strain and laboratory strain) in different media at different temperatures (15°C, 23°C, 29°C, 37°C).

Sample type	pH value of sample	Temperature [°C]	Maximal duration after which cultivation of leptospires was successful [h]	
			Laboratory strain—*L*. *kirschneri* serovar Grippotyphosa (Strain Moska)	Field strain—*L*. *kirschneri* serovar Grippotyphosa (LA1-RoBo-Pub)
Cattle urine undiluted	8.4	15	0	0
		23	0	0
		29	0	0
		37	0	0
Dog urine undiluted	5.8	15	0	0
		23	0	0
		29	0	0
		37	0	0
Cattle urine diluted in PBS (1:10)	7.7	15	4	4
		23	4	4
		29	4	2–4
		37	1	2
Cattle urine diluted in purified water (1:10)	7.3	15	48–72*	4
		23	24	4
		29	4	4
		37	2	4
Dog urine diluted in PBS (1:10)	6.7	15	4	4–24*
		23	2	4
		29	2	4
		37	2	4
Dog urine diluted in purified water (1:10)	5.5	15	4	4–24*
		23	4	4
		29	4	4
		37	2	4
PBS	7.3	15	≥ 24**	≥ 24**
		23	≥ 24**	≥ 24**
		29	≥ 24**	≥ 24**
		37	≥ 24**	≥ 24**
EMJH medium	7.4	15	≥ 168**	≥ 168**
		23	≥ 168**	≥ 168**
		29	≥ 168**	≥ 168**
		37	≥ 168**	≥ 168**

*Cultivation attempts were done in triplicate—in 1 of three tubes a cultivation attempt was successful at the last time point

** last time point tested

All positive controls at all temperatures showed leptospiral growth and all cultivation attempts of leptospires in culture stored at different temperatures were successful (Table 1).

### Distribution and survival of *L*. *kirschneri* serovar Grippotyphosa in a water stream

Leptospires were detected with the real-time PCR at all time points (Fig 1, X1-X6). However, cultivation attempts failed for all samples (laboratory and field strain). In contrast, in the control experiment conducted with stagnant tap water, the cultivation of leptospires was possible for all samples.

### Stability of *L*. *kirschneri* serovar Grippotyphosa while drying

At all tested temperatures it was possible to cultivate leptospires at the time point when the culture was almost dried on the steel discs. That was after 90 min at 15°C, 60 min at 23°C, and 30 min both at 29°C and 37°C. After complete drying, which was exactly 30 min after the time points just mentioned, it was impossible to cultivate leptospires at any temperature. The results of the experiments with the field strain of *L*. *kirschneri* serovar Grippotyphosa and the laboratory strain did not differ.

## Discussion

### Survival of *L*. *kirschneri* serovar Grippotyphosa in animal urine

In this study, we tested and compared the survival time of a laboratory and a field strain of *L*. *kirschneri* serovar Grippotyphosa under different environmental conditions. It has been described that freshly isolated pathogenic *Leptospira* species are usually shorter and more tightly coiled than strains that have undergone more than 20 passages in a laboratory [31, 32]. This morphological change is often connected to decreased motility and “poor cell health” [32]. Our results demonstrated that both tested strains of *L*. *kirschneri* serovar Grippotyphosa were not able to survive in undiluted cattle or dog urine even for the shortest period tested, i.e. 1 min. The few earlier studies examining the survival of *Leptospira* spp. in animal urine used different study conditions, making a direct comparison of our results difficult. In a study conducted by Khairani-Bejo et al. [18] *L*. *interrogans* serovar Hardjo survived 0–6 h at similar temperatures in undiluted cattle urine. The reason for these non congruent findings could be the tested leptospiral species, their adaptation to different animals or the capability to resist possible harmful effects of the urine. In contrast to serogroup Grippotyphosa, the main reservoir of serogroup Hardjo is cattle [33]. Also, a different biochemical composition of the urine used may have influenced survival time [34]. It has been described that feeding habits, physical activities, body size, and even the climate of the resident location alter the chemical composition of cattle urine [34]. Another reason for the different survival time could be the distinct methods used to determine the endpoint of survival in the study of Khairani-Bejo et al. [18] and ours. In our research, the survival time was defined as the longest period after which the cultivation in the modified EMJH medium was possible. Khairani-Bejo et al. [18] tested hamsters for leptospirosis after inoculation and equated the survival time with the latest time point when leptospires were still infectious. Whilst there are so many variables, it is hard to reliably compare the results of in vivo and in vitro tests. As a congruency of in vitro test results and results of experimental infections regarding the viability of the bacteria has not yet been proven, further tenacity studies should focus on the comparability of these test methods.

In any case this suggests that contaminated urine needs fast dilution in order to guarantee leptospiral survival. Among animals shedding *Leptospira* spp. adapted to this particular host species, this may differ. Here, a direct infection route through for example sniffing, licking and maybe grooming may make a long survival in urine unnecessary.

In diluted cattle urine, the laboratory strain of *L*. *kirschneri* serovar Grippotyphosa was able to survive for 72 h (maximum tested period of 7 days) at 15°C. This observation of maximal survival time at the lowest tested temperature is in accordance with the findings of Khairani-Bejo et al. [18]. In their study, *L*. *interrogans* serovar Hardjo survived the longest in diluted cattle urine at 4°C (48–984 h) and had the shortest survival time at the highest temperature tested (48 h at 32°C) [18].

In the current study, the laboratory strain survived longer in diluted cattle urine with an almost neutral or slightly alkaline pH of 7.3–7.7 which is in line with the description of an optimal pH range for leptospiral growth of 7.2–7.6 [32]. In contrast, the field strain of L. grippotyphosa survived the longest in diluted dog urine with a more acidic pH value of 5.5–6.7. *Leptospira kirschneri* serovar Grippotyphosa is often the causative agent of canine leptospirosis hence these differences could occur from an adaption of the field strain to the acidic urine of dogs (pH < 7), whereas the laboratory strain may have lost this ability by being maintained for many life cycles in special medium with a pH value of 7.4 [13, 32].

The longer survival of both strains in a colder environment could be explained by harmful reactions of enzymes present in the urine and their increased activity at higher temperatures [35]. Another possible explanation may be the precipitation of salts, like struvite in the urine at lower temperatures [36]. Despite some exceptions, *Leptospira* spp. have been described to be susceptible to higher concentrations of salts in the environment [18, 37, 38]. A study by Albasan et al. [36] showed a greater crystal size of salts in animal urine stored at lower temperatures. This increased size could result in a faster descent of these salt particles and therefore a lower likelihood of contact between salt crystals and leptospires, which are normally found on the upper surface of fluids. The long maintenance of the laboratory strain in an optimized medium without harmful substances for the leptospires could lead to a possible loss of their capability to resist them. It may also explain the different influences of temperature between the field and the laboratory strain [32]. This loss of resistance and the previously described susceptibility to salt is also a probable explanation for the differences in survival time of the laboratory strain depending on the dilution of the urine in either PBS or purified water. In all experiments conducted with animal urine, a decreased viability could be the result of the residual urine in the EMJH medium during the incubation period.

### Distribution and survival of *L*. *kirschneri* serovar Grippotyphosa in a water stream

In consequence of the necessity to dilute the urine, we investigated how efficient leptospires might be transported in a water current. Leptospires were detected by PCR at all time points tested in the 50-meter-long hose system. Although the detection of the *Lipl 32* gene by real-time PCR does not necessarily mean that there were still live *Leptospira* cells in the tested samples, these results suggest that the leptospires were not only dragged with the water stream at the same speed but also traveled faster and slower than the average velocity (0.1 m/s) of the water. These findings are explainable by the intrinsic mobility of *Leptospira* spp. either in the same or against the direction of the water stream. Our data are in line with the experimental findings of Okazaki and Ringen where migration of leptospires up a slow-moving stream was suggested [19]. The speed of leptospires has been described to be dependent on the viscosity of the surrounding medium and is approximately 20 μm in 2–3 sec in regular medium [39]. These findings could be fundamental in risk assessment for leptospiral outbreaks.

As the cultivation of leptospires from the hose system was not possible in contrast to leptospires kept in stagnant water (for the same time), mechanical damage of the leptospires in the hose system is the most likely explanation. Although the use of the 0.22 μm filter could have also reduced or eliminated leptospiral cells in the samples and therefore be responsible for the failed cultivation attempts, mechanical damage seems to be a more likely explanation, as also the leptospires kept in stagnant water were filtrated the same way and cultivation was possible afterwards. Hence, we postulate that the survivability of leptospires could increase when excreted into stagnant water in the environment, but excretion of *Leptospira* spp. into a stream with high velocity in nature could be harmful to *Leptospira* spp., because of a greater chance of mechanical impairment.

### Stability of *L*. *kirschneri* serovar Grippotyphosa while drying

*Leptospira kirschneri* serovar Grippotyphosa was not able to survive complete drying on a surface in our experiments. Other studies described a correlation between the humidity of the surroundings and the viability of leptospires [18, 19, 40]. For example, Karaseva et al. showed increasing survival times of leptospires connected with rising moisture in the soil, which suggests that leptospiral survival in the environment depends on a sufficient level of humidity [40].

## Conclusions

In this study *L*. *kirschneri* serovar Grippotyphosa survived up to three days in diluted animal urine and did not survive in undiluted cattle or dog urine. Therefore, the most crucial point regarding the survivability of leptospires seems to be a fast dilution in the environment after having been excreted via urine or a direct intake of infected urine by naїve animals. Comparing different temperatures both strains survived longer in diluted animal urine at lower temperatures. *Leptospira kirschneri* serovar Grippotyphosa did not survive drying on a solid surface. Hence, lower temperatures, as well as humid environments, appear to prolong the tenacity of leptospires against detrimental influences, while drought does not allow survival of the leptospires. In a water stream, leptospires were able to move faster or slower than the average velocity of the water due to their intrinsic mobility but were not able to survive the mechanical damage caused by running water in the hose system. Thus, a dilution in stagnant water or a slow stream without mechanical damage could favor the survival of viable bacteria and due to their proper motion, leptospires are likely to spread from the place of their excretion. However, the speed, distance of their spread and survival have to be investigated further. Our findings show differences in the survivability between strains from the laboratory and the field. This suggests that future studies should focus not only on the cultures maintained in the laboratories but also on freshly isolated *Leptospira* spp.
